# HFE Genotyping in Patients with Elevated Serum Iron Indices and Liver Diseases

**DOI:** 10.1155/2015/164671

**Published:** 2015-01-14

**Authors:** Andreia Silva Evangelista, Maria Cristina Nakhle, Thiago Ferreira de Araújo, Clarice Pires Abrantes-Lemos, Marta Mitiko Deguti, Flair José Carrilho, Eduardo Luiz Rachid Cançado

**Affiliations:** ^1^Department of Gastroenterology from University of Sao Paulo School of Medicine, Avenue Dr Eneas de Carvalho Aguiar 255, 05403-000 Sao Paulo, SP, Brazil; ^2^Laboratory of Medical Investigation (LIM-06), Institute of Tropical Medicine, University of Sao Paulo, Sao Paulo, SP, Brazil

## Abstract

Iron abnormalities in chronic liver disease may be the result of genetic diseases or secondary factors. The present study aimed to identify subjects with HFE-HH in order to describe the frequency of clinical manifestations, identify risk factors for iron elevation, and compare the iron profile of HFE-HH to other genotypes in liver disease patients. A total of 108 individuals with hepatic disease, transferrin saturation (TS) > 45%, and serum ferritin (SF) > 350 ng/mL were tested for HFE mutations. Two groups were characterized: C282Y/C282Y or C282Y/H63D genotypes (*n* = 16) were the HFE hereditary hemochromatosis (HFE-HH) group; and C282Y and H63D single heterozygotes, the H63D/H63D genotype, and wild-type were considered group 2 (*n* = 92). Nonalcoholic liver disease, alcoholism, and chronic hepatitis C were detected more frequently in group 2, whereas arthropathy, hepatocarcinoma, diabetes, and osteoporosis rates were significantly higher in the HFE-HH group. TS > 82%, SF > 2685 ng/mL, and serum iron > 178 *μ*g/dL were the cutoffs for diagnosis of HFE-HH in patients with liver disease. Thus, in non-Caucasian populations with chronic liver disease, HFE-HH diagnosis is more predictable in those with iron levels higher than those proposed in current guidelines for the general population.

## 1. Introduction

Elevated serum iron can occur in a variety of conditions, such as chronic liver diseases, viral hepatitis, alcoholic and nonalcoholic disease, hematologic processes with ineffective erythropoiesis, hemolytic anemias, and transfusional iron overload. Elevated serum iron may also denote a genetic condition, and hereditary hemochromatosis (HH) is the best described primary iron-related disorder.

Traditionally, HH has been characterized by elevated serum biochemical markers and parenchymal iron with resultant diabetes, hepatic cirrhosis, and skin hyperpigmentation. In 1996, discovery of the HFE gene highlighted the physiopathology of iron metabolism [1]. Mutations in this gene are Celtic in origin and are distributed according to their migration pattern [2, 3]. Allele frequencies are higher in northern Europe and lower, or even absent, in southern Europe [4]. Caucasians with HH have been recognized as carriers of* HFE* mutations, with 90–95% carrying homozygous C282Y mutations and 3–5% being compound heterozygous for C282Y/H63D [1, 4, 5]. The other genotypes, such as single C282Y and H63D heterozygotes or H63D homozygotes, are related to mild elevations in iron, especially when associated with risk factors [5]. Knowledge about HH physiopathology and its diagnosis has evolved; other hereditary forms have been described and classic manifestations have been recognized to be a result of advanced stage iron deposition. Subjects have been identified in the early stages of disease.

Before 1996, many cases of iron overload diagnosed as HH were revised, and some previously described as heterozygotes because of the mild nature of symptoms and clinical manifestations were, indeed, homozygous in the early stages of disease. Despite this known clinical picture, the phenotypic definition of HH is difficult because of the extreme variability (1–28%) in* HFE* gene penetrance [4]. Although approximately 80% of C282Y homozygotes present with elevated serum iron levels, a poor correlation exists with significant disease, and it is not possible to predict which homozygous carriers will develop clinically significant disease [6, 7]. Risk factors are important contributors to the expression of the disease or, in the absence of genetic predisposition, the only cause of iron overload [8, 9].

The concept of iron overload-related disease was first introduced to define patients with iron overload in the blood and tissue associated with liver disease, hepatocellular carcinoma (HCC), and arthropathy [10]. The prevalence of other manifestations attributed to HH is the subject of controversy in studies. Elevated iron indices associated, or not associated, with iron overload may be present in innumerous conditions, and differential diagnosis is challenging. Moreover, chronic liver disease may be the only identified because of increased iron in subjects, even in the absence of HH. Identifying HH from among the biochemical abnormalities associated with iron, the associated clinical manifestations, and other underlying factors may avoid unnecessary treatment and additional morbidity in patients. In addition, global allele frequencies are even lower than those demonstrated in northern European populations. Thus, HH diagnosis is challenging given the diversity of confounding or contributing factors that could lead to higher iron levels [11]. In Brazil, a country of admixed people, allele frequencies are lower than those found in Caucasians, and HH diagnosis is made in only 50% of individuals with iron overload [12].

The aim of this study was to identify subjects with HFE-HH, characterize and describe frequency of clinical manifestations, identify associated risk factors for iron elevation, and compare the iron profiles of patients with the HFE-HH genotype versus other genotypes in a Brazilian population of patients with liver disease and elevated serum iron indices.

## 2. Methods

Over a period of 3 years, patients from the Center of Hepatology of Hospital das Clinicas, a tertiary Hospital of Sao Paulo University in Brazil, were referred to search for* HFE *mutations for presumed iron overload. Patients who presented with transferrin saturation (TS) > 45% and serum ferritin (SF) > 350 ng/mL were considered for inclusion in this study. Exclusion criteria were acute hepatitis, absence of clinical data, and unavailability of patient DNA. Patient records were analyzed for clinical manifestations, risk factors for iron overload, liver biopsies, liver imaging methods, and biochemical iron markers. Hepatic cirrhosis was identified with grade 4 fibrosis in liver biopsies or the presence of signs of chronic hepatopathy in the physical examination (e.g., ascitis, palmar erythema, asterixis, and collateral veins) or complimentary analysis, such as abdominal imaging or laboratory measures (e.g., low platelet levels, low albumin, elevated bilirubin, and prolonged PT). Documented serum iron indices were TS, SF, serum iron (SI), transferrin, and total iron binding capacity (TIBC).* HFE* mutations C282Y, H63D, and S65C were screened by restriction fragment length polymorphism polymerase chain reaction (PCR-RFLP). The diagnosis of classical hereditary hemochromatosis (HFE-HH) was established for genotypes C282Y/C282Y and C282Y/H63D. HFE-HH patients were compared to the remaining cases: genotypes C282Y/−, H63D/−, H63D/H63D, and the absence of* HFE *mutations (wild-type). Statistical analyses were performed using R software, 2.15.2 version. Biochemical markers were compared by the Mann-Whitney and* t*-test, and Fisher's test was used to verify the association among categorical variables. Analysis of variance (ANOVA) was used to analyze more than two groups, and logistic regression with the “backward method” was used to associate* HFE* genotypes and clinical manifestations. Cutoffs were defined by a receiver operating characteristic (ROC) curve using the Youden method. The significance level was set at *P* < 0.05. This study was approved by the local ethics committee of our hospital, and all patients provided signed informed consent.

## 3. Results

A total of 231 patients were referred for* HFE* genotyping, 133 of whom fulfilled the inclusion criteria. Twenty-five patients were subsequently excluded, 1 due to a clinical diagnosis of acute hepatitis and 24 due to missing clinical data. Therefore, 108 patients were analyzed. Based on the genotyping results, 16 of the patients were diagnosed as HFE-HH: 13 were homozygous for C282Y and 3 were compound heterozygous for C282Y/H63D. The non-HFE-HH iron elevation group comprised 92 patients that were negative for HFE-HH genotypes: 54 had no* HFE* mutation and 38 had at least one* HFE* mutation (C282Y +/−, *n* = 7; H63D +/−, *n* = 27; H63D +/+, *n* = 4). None of the tested individuals carried the S65C mutation. Two of the patients in the non-HFE-HH iron elevation group were siblings, and four patients with HFE-HH comprised a pair of cousins and a pair of siblings.

The mean age for all patients was 46.7 years (range: 16–77 years), 77.6% of patients were male, and 70.4% were Caucasian. Risk factors for serum iron elevation were nonalcoholic fatty liver disease (NAFLD; 34.6%, 35/101), alcoholic liver disease (ALD; 26%, 27/104), and chronic hepatitis C (24.8%, 26/105). Among all patients in the study, 64.8% (*n* = 70) were cirrhotic according to the criteria predefined in the methods. The characteristics of both groups are provided in Table 1.

### 3.1. Iron in Chronic Liver Disease

**Figure 1:**
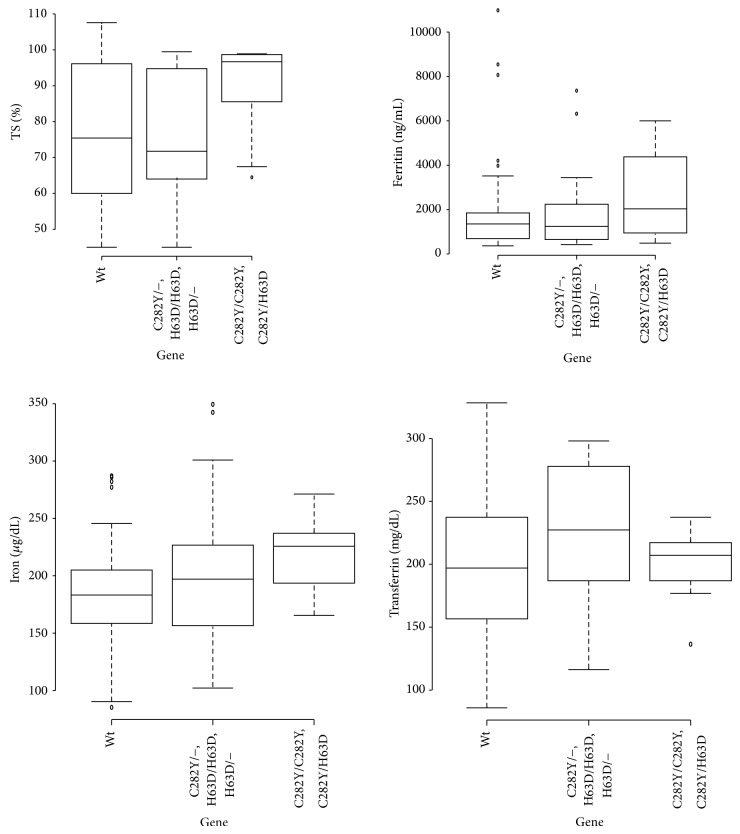
Biochemical iron markers and relationship with HFE genotype. Higher levels of transferrin saturation were observed in the HFE-HH genotype when compared to other HFE genotypes and wild type (96.71% versus 71.75%, *P* = 0.002, and 96.71% versus 75.44%, *P* = 0.004, resp.). Significant differences were also observed among serum iron levels (221 *μ*g/dL, 192 *μ*g/dL and 178 *μ*g/dL, resp., *P* = 0.008). Nonsignificant differences in ferritin levels were observed between HFE-HH genotype group, and other HFE genotypes and wild type (3323.50 ng/mL, 1246.5 ng/mL, and 1354.5 ng/mL, resp., *P* = 0.192). Furthermore, nonsignificant differences regarding the median of transferrin levels in each group were observed (200 mg/dL, 220 mg/dL, and 190 mg/dL, resp., *P* = 0.201).

Liver biopsies were performed in 74 patients. Grade 3 and 4 fibrosis were present in 9 (12.2%) and 36 (48.6%) patients, respectively. Results were available for Perls' staining analysis in 69 liver tissue sections, including 10 from the HFE-HH group. The HFE-HH group had higher siderosis grades than the non-HFE-HH iron elevation group (*P* = 0.026, Table 2). Thirteen patients with HFE-HH and 30 patients in the non-HFE-HH iron elevation group underwent phlebotomy. The iron indices in both groups are provided in Table 2, and their distributions according to genotype are shown in Figure 1.

### 3.2. Iron Overload

**Figure 2:**
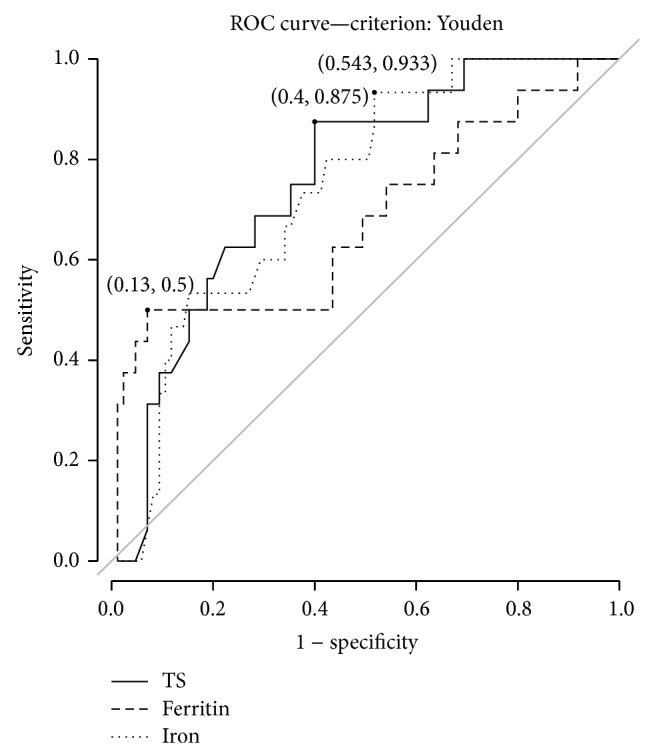
ROC curve for biochemical iron markers TS, serum ferritin and iron and diagnosis for HFE-HH genotypes. The cutoff values for the diagnosis of HH were TS > 82%, serum ferritin levels >2685 ng/mL, and serum iron levels >178 *μ*g/dL. The areas under the curve (AUC) were 0.734, 0.643, and 0.719, respectively. It is important to stress that TS showed a higher accuracy when compared to serum iron levels. Conversely, ferritin presents with the lowest accuracy, as demonstrated by the lower AUC.

According to the concept of iron overload-related disease, 10 patients in the non-HFE-HH iron elevation group had high serum iron levels, siderosis grades 3 and 4 on liver biopsy, and liver disease. Presumably, these patients have true iron overload. However, six of these patients did not have any risk factor for iron overload, but they possibly had another type of HH. After excluding these six patients from the analysis, TS, serum iron, and SF were higher in patients with HFE-HH than those remaining in the other group (Table 2). The cutoffs for HFE-HH diagnosis were TS > 82%, SF > 2685 ng/mL, and serum iron > 178 *μ*g/dL. The ROC curves are shown in Figure 2.

### 3.3. Clinical Manifestations

Arthropathy, HCC, osteoporosis, and diabetes were more frequent in the HFE-HH group than the non-HFE-HH iron elevation group (Table 3). In multiple regression analysis, HCC remained the only variable associated with HFE-HH genotypes (OR = 5.81, *P* = 0.018). Compared to the subgroups wild-type and other HFE mutations, patients with HFE-HH genotypes were more likely to develop HCC (OR = 5.0, *P* = 0.032).

The proportion of patients without an additional risk factor for liver disease (other than iron overload) was 37.5% in the HFE-HH group and 11.0% in the non-HFE-HH iron elevation group (*P* = 0.019).

## 4. Discussion

In a cohort of patients with liver disease and elevated iron indices, 26 patients presented evidence of a primary iron overload-related disease. Among these patients, more than half (61%) carried HFE-HH genotypes. In six of the remaining 10 patients, no comorbidities or risk factors for iron overload were detected, and other hereditary forms of hemochromatosis may be present. Three of these subjects did have clinical characteristics of juvenile hemochromatosis. A homozygous G → A mutation at position +14 of the 5′ untranslated region (5′UTR)of* HAMP* [13] was detected in three patients, including two siblings and one unrelated patient. An investigation of other mutations related to non-HFE HH is currently in progress and does not constitute part of the main objective of the current study.

In Brazil, the prevalence of* HFE* mutations is lower than in populations of northern Europe. In a previous Brazilian report, allelic frequencies of 1.4%, 1.1%, 1.1%, and 0% were reported for the C282Y polymorphism in Caucasians, subjects of African descent, racially mixed subjects, and Amerindians, respectively [14]. The H63D mutation was observed at higher allelic frequencies in all populations but, as reported for C282Y, was 0% in Amerindians, possibly reflecting more dissemination in populations around the world and no distribution in Amerindians. In an analysis of blood donors, the allelic frequency of the C282Y mutation was 0.4% and disease frequency of HFE-HH 0.1% [15]. In European populations, the allelic frequency of C282Y has been reported to be 6.2%, with significant geographic differences, ranging from 12.5% in Ireland to 0% in southern Europe [4]. The Brazilian population is racially diverse due to a heterogeneous ethnic origin. Immigrants from European and African countries and Japan, as well as the native population, are admixed, which could explain the variability, diluting or eliminating* HFE *mutations.

In Brazilian patients with high iron indices and liver disease, the frequencies of HFE-HH genotypes were very low, as suggested in previous reports [12, 16]. In this study, more than a half (60%) of 26 patients with an iron overload-related disease, elevated serum iron levels, liver disease, and advanced siderosis were identified as carriers of HFE-HH genotypes (C282Y/C28Y and C282Y/H63D), with 50% C282Y homozygosity (*n* = 13/26). This finding is in line with a previous report that homozygous C282Y is present in 47% of patients with the HH phenotype and suggests that other mutations could play an important role in Brazilian patients with HH [12]. However, among patients with variable liver disease, only 24% presented with iron overload (26/108), and in 14% (16/108) the genotype was characteristic of HFE-HH. This finding could reflect that, as expected, not all patients who exhibited high iron indices had iron accumulation in tissues, and the lower frequencies of HFE-HH genotypes may be in line with what was previously shown in the Brazilian population. Here, the absence of comorbidities was demonstrated in only 37.5% of patients with HFE-HH. Environmental factors play an important role in HFE-HH disease expression [8, 9], and this could be relevant in an admixed population such as that of Brazil. The high frequencies of comorbidities could reflect a bias because the subjects were selected from a liver disease center.

The real association of the so-called classical manifestations of HH with HFE genotypes is very difficult to assess by the reports because of the variability in the clinical presentation of this disease [4]. EASL guidelines recommend considering screening for HH in patients with unexplained chronic liver disease and those with cutanea tarda porphyria. Other recommendations are HCC, type 1 diabetes, and well-defined chondrocalcinosis due to higher prevalence of C282Y homozygosity in these patients [4]. Dever et al. demonstrated a higher proportion of impotence, diabetes mellitus, and hypothyroidism in HH patients, though without reaching significance [17]. The association of diabetes type 2, arthropathy, and C282Y polymorphism has not been established, but carriers of HH with advanced disease may present with a higher proportion of these clinical manifestations [4]. Patients with cirrhosis, arthropathy, and diabetes represent the morbidity associated with clinically manifest disease in HH and are classified in the fourth stage of the scale of HH phenotypic expression [18]. In our study, diabetes, osteoporosis, and arthropathy were detected more frequently in the HFE-HH group, which would lead one to expect clinical findings of disease in those with this genotype. Therefore, the diagnosis of HFE-HH could be facilitated when one or all of these morbidities are present in a patient with liver disease and high iron levels. The selection of patients with liver disease, and in the majority of cases cirrhosis, could favor this scenario because, as our patients had evidence of advanced disease, they are presumably included in stage 4 of that classification in which the probability of finding these clinical manifestations is greater. On the other hand, these findings are also more frequent in patients with liver disease, and a larger sample size is required to demonstrate the real association of these comorbidities with HFE-HH genotypes.

Carriers of HFE-HH genotypes have a higher risk of developing HCC compared to those with the wild-type genotype or one* HFE* mutation [4]. The frequency of C282Y homozygosity was shown to be 5.5–10% in patients with HCC in one report, whereas other authors observed an increased prevalence of C282Y mutation [4]. The studies of C282Y frequency in patients with HCC present limitations in their analysis due to small samples. In these studies, the etiology of HCC was extremely variable [4], and their conclusions require additional confirmation. The risk of developing HCC seems to be higher in patients with HH [19–21]. In multiple regression analysis, HCC was the only variable associated with the HFE-HH group. Considering the majority of patients with hepatic diseases of different etiologies, HFE-HH seems to be the main cause implicated in the emergence of HCC. This finding is relevant because it strengthens HCC as a remarkable condition that should be screened cautiously in patients with HFE-HH, and it brings to light the role of* HFE* genotypes in the development of this comorbidity. Despite these findings, the number of cases in the present study is small, and a higher number of patients could result in more definitive conclusions.

Analysis of the biochemical iron profile suggests that the iron levels are higher in patients with liver disease than in controls, particularly among HFE-HH subjects [17, 22, 23]. In our study, HFE-HH genotypes were unlikely to be found in patients with liver disease and TS < 82%. Also, TS was higher in those with HFE-HH genotypes compared to those with the wild-type genotype or one* HFE* mutation. Despite the low number of patients with HFE-HH, this result is supported by reports that TS > 50% or 60% is a reliable predictor of HFE-HH in liver clinics [22, 24]. Patients with liver diseases also may present with higher TS due to a decrease in transferrin levels or hemolysis secondary to a portosystemic shunt; thus, Nichols et al. [25] suggested that TS is not a reliable marker for diagnosing HFE-HH in patients with liver disease. In contrast, in the general population, the threshold recommended starting screening for HH should be 45% TS [4, 5, 26]. Our study does not address testing the general population, as the patients were selected for advanced liver disease. Our population profile may explain the higher TS compared to other studies. HFE-HH should be the first suspicion in patients, especially non-Caucasians, with chronic liver disease and TS > 82%. Lower levels in this population suggest that other factors elevating iron levels should be investigated.

The serum ferritin level is a good predictor of the presence of advanced fibrosis. A ferritin level >1,000 ng/mL in HH is associated with advanced fibrosis and disease severity [26–29]. In our study of patients with advanced disease, the serum ferritin cutoff for the diagnosis of HFE-HH mutation was high at 2,685 ng/mL. The elevated specificity for HFE-HH mutation diagnosis in our study may reflect that the HFE-HH genotype results in higher iron levels and, as shown previously, serum ferritin concentrations <1,000 ng/mL are at low risk of developing HH-associated signs and symptoms [30]. As our patients are carriers of liver disease, and the majority cirrhotic, ferritin values <1,000 ng/mL are unlikely to be found. The loss of accuracy could be explained by the fact that, as a nonspecific marker present in many conditions not necessarily related to iron overload, this marker may occur at extremely high levels in our population comprised predominantly of cirrhotics, with several cofactors that could increase ferritin levels [31]. The high level of ferritin necessary for HFE-HH diagnosis is possibly explained by the underlying liver disease, but it can also reflect a great number of comorbidities revealed in the groups. The best approach is probably to combine both analyses, that is, TS and ferritin, before searching for* HFE* mutations in liver disease populations. Serum iron levels presented high sensitivity for HFE-HH genotype diagnosis, but a lower specificity and predictive positive value compared to TS. In addition, marked variability in serum iron levels throughout the day limits this test for detecting hemochromatosis, and it is not different for our population of patients with liver diseases of diverse etiologies [24].

The number of patients with HFE-HH is a limiting factor in our work. Another limitation is the number of comorbidities assessed in the groups, such as osteoporosis and cardiopathy, which is characteristic of retrospective studies. However, this does not seem to affect the main objective and conclusions of this study. In the Brazilian population, finding a large number of HFE-HH patients is challenging given the low frequency of* HFE* mutations. Nevertheless, this study highlights the differences in iron levels in patients with liver diseases of different etiologies and the association with HFE-HH. In addition, and most importantly, this study suggests to the clinician the most suitable time to screen for HFE-HH in a non-Caucasian liver disease population. Knowledge about the behavior of this illness in Brazil is necessary for adequate diagnosis and support.

## 5. Conclusion

This study focused on the differential diagnosis of patients with liver disease and altered serum iron indices. Frequently, patients with high serum iron indices are misinterpreted as having HH. In the age of molecular biology and mutation screening, typing patients for HH mutations is the first choice for diagnosing this condition. However, the appropriate time to search for HFE mutations in patients with liver diseases is still a challenge for clinicians, especially when other diagnoses are present. This study shows that, in non-Caucasian patients with liver disease, few patients present with iron overload and HFE-HH is responsible for approximately half of cases with iron overload. Therefore, other mutations and conditions that lead to iron overregulation should be investigated. In patients with liver diseases, different cutoffs for biochemical iron tests are expected than those established for the general population, with higher levels detected before testing* HFE* mutations. In patients with liver disease and HFE-HH genotypes, HCC is the comorbidity worth highlighting in the scenario of complications that can arise in patients with stage 4 disease and should be systematically screened.

## Figures and Tables

**Table 1:** Characteristics of patients.

Characteristic	HFE-HH (*n* = 16)	Non-HFE-HH iron elevation (*n* = 92)
Age, mean ± SD	51.07 ± 9.6	45.92 ± 13.65
Male, *n* (%)	12 (75.0)	72 (78.26)
Caucasoid, *n* (%)	11 (68.75)	65 (70.65)

Risk factors for elevated iron indices		
Nonalcoholic liver disease, *n*/*N* (%)	3 /16 (18.75)	32/85 (37.65)
Alcoholic disease, *n*/*N* (%)	2/15 (13.3)	25/89 (28.09)
Chronic hepatitis C, *n*/*N* (%)	1/16 (6.25)	25/89 (28.09)
Porphyria cutanea tarda, *n*/*N* (%)	0/16 (0)	1/92 (7.69)
Human immune deficiency virus, *n*/*N* (%)	0/16 (0)	4/92 (4.35)
Chronic hepatitis B, *n*/*N* (%)	0/16 (0)	2/88 (2.27)
Chronic kidney disease, *n*/*N* (%)	0/16 (0)	2/90 (2.22)

HFE-HH: HFE hereditary hemochromatosis.

Non-HFE-HH iron elevation: patients with iron elevation not related to HFE-HH genotypes.

*n* = number of patients affected.

*N* = total number of individuals assessed.

**Table 2:** Differences between siderosis and biochemical iron markers.

Liver tissue analysis (Perls stain)	HFE-HH *n* = 10	Non-HFE-HH iron elevation *n* = 59	
Siderosis grade			
0	0	15	
1	0	13	
2	1	10	
3	5	8	
4	4	11	

Serum iron indices	*n* = 16	*n* = 86	*P* value

TS, %	91.02 ± 11.26	75.56 ± 17.59	0.001
Serum iron, mg/dL	212.65 ± 30.82	180.13 ± 48.16	0.001
Serum ferritin, *μ*g/L	2676.75 ± 1928.87	1366.85 ± 945.88	0.012
Transferrin, mg/dL	192.73 ± 27.96	203.37 ± 57.47	0.830
Total iron binding capacity	229.46 ± 29.65	252.81 ± 78.26	0.975

HFE-HH: HFE hereditary hemochromatosis.

Non-HFE-HH iron elevation: patients with iron elevation not related to HFE-HH genotypes.

Results are expressed as mean ± SD.

Results present a comparison of the biochemical profiles of both groups after excluding six probable non-HFE HH from the analysis.

**Table 3:** Clinical manifestations in patients.

Clinical manifestation	HFE-HH (*n* = 16)	Non-HFE-HH iron elevation (*n* = 92)	*P* value
Arthropathy	7/13 (53.8)	7/44 (15.9)	0.008
Cardiopathy	10/14 (71.4)	23/36 (63.8)	0.500
Cirrhosis	12/16 (75.0)	58/92 (63.0)	0.316
Hepatocellular carcinoma	5/16 (31.2)	6/85 (7.06)	0.008
Skin hyperpigmentation	4/10 (40.0)	15/55 (27.2)	0.331
Diabetes	9/16 (56.2)	27/90 (30.0)	0.040
Hypogonadism	5/13 (38.4)	9/61 (14.7)	0.056
Osteoporosis	8/11 (72.7)	9/28 (32.1)	0.026
Thyroidopathy	4/16 (25.0)	10/88 (11.3)	0.142

Data are presented as number of patients affected/total number of individuals assessed (%).

HFE HH: HFE hereditary hemochromatosis.

Non-HFE-HH iron elevation: patients with iron elevation not related to HFE-HH genotypes.
